# Retraction: MicroRNA-218 inhibits proliferation and invasion in ovarian cancer by targeting Runx2

**DOI:** 10.18632/oncotarget.28253

**Published:** 2022-07-02

**Authors:** Na Li, Lufei Wang, Guangyun Tan, Zhiheng Guo, Lei Liu, Ming Yang, Jin He

**Affiliations:** ^1^Department of Gynecology and Obstetrics, The First Hospital of Jilin University, Changchun, Jilin 130021, PR China; ^2^Department of Ophthalmology, The Second Hospital of Jilin University, Changchun, Jilin 130022, PR China; ^3^Department of Immunology, Institute of Translational Medicine of The First Hospital of Jilin University, Changchun, Jilin 130021, PR China; ^4^Department of Breast Surgery, The First Hospital of Jilin University, Changchun, Jilin 130021, PR China; ^*^These authors have contributed equally to this work

**This article has been retracted:** Oncotarget has completed its investigation of this paper. We found that Figures 2C, 2E, 5C, 5D, 6E, and Table 1 contain similarities with data appearing in other articles by different authors. The data had already been published elsewhere, or were already under consideration for publication prior to submission to Oncotarget. During our long period of communication with the authors, we received a request from corresponding author Jin He to retract the paper. As of this writing, Oncotarget has not received signed documentation from all authors acknowledging the retraction. Oncotarget has also notified the affiliated institution regarding this retraction.

Original article: Oncotarget. 2017; 8:91530–91541. 91530-91541. https://doi.org/10.18632/oncotarget.21069

